# The complete mitochondrial genome of the soldier fly *Ptecticus aurifer*

**DOI:** 10.1080/23802359.2020.1711820

**Published:** 2020-01-16

**Authors:** Qingbin Zhan, Yang Zhao, Siyu Zhang, Xujian Peng

**Affiliations:** a Nanjing Forest Police College, Nanjing, China;; b Key Laboratory of Wildlife Evidence Technology State Forest and grassland Administration, Nanjing, China;; c Nanjing institute of Agricultural Science, Nanjing, China

**Keywords:** Mitochondrial genome, forensic insect, *Ptecticus aurifer*

## Abstract

The pig body was put in the wild area in summer for collecting sarcosaphagous insects. After 31-day (9 June 2015 to 9 July 2015), the pig body was to be mummification. *Ptecticus aurifer* (Walker 1854) was found as sarcosaphagous insect for the first time. The complete mitochondrial genome of *P. aurifer* (Walker 1854) was sequenced in this study. The complete mitochondrial genome is a typical double-stranded circular molecule of 15,775 bp (GenBank accession number: MN604259) containing 37 typical animal mitochondrial gene and an A + T-rich region. 11 of the 22 tRNAs, ranging from 63 to 72 bp, can be folded into classic clover-leaf secondary structure except for *tRNA^Ser(AGN)^*, in which the dihydrouridine (DHU) arm did not form a stable stem-loop structure. The control region is 954 bp long with an A + T content of 90.7%.

Stratiomyidae, the soldier flies, are a diverse family of orthorrhaphous Brachycera. There are 375 extant genera of Stratiomyidae, arranged in 12 subfamilies. The Neotropical Region contains the highest stratiomyid species diversity of all the biogeographic regions (Woodley 2001).

Though they exhibit great morphological variation, the family is distinct from other flies due to their unique wing venation and larval habitus. The adults are most often collected on foliage in damp forests, near bodies of water, or near boggy areas (James 1981). Many genera, particularly in the Stratiomyinae and Nemotelinae, visit flowers. Some soldier flies exhibit interesting mating behaviors, such as *Hermetia pterocausta,* males of many species of Beridinae, Pachygastrinae and *Microchrysa*. They can form large mating swarms (Woodley 2001).

After 31-day (9 June 2015 to 9 July 2015), the pig body was to be mummification. The insect community succession was also observed, and *Ptecticus aurifer* (Walker 1854) was found as sarcosaphagous insect for the first time stored in the key laboratory of wildlife evidence technology state forest and grassland administration (specimen code NFPC8812). In this study, we present the complete mitochondrial genome of *P. aurifer* (Walker, 1854).

This mitochondrial genome is 15,775 bp long (GenBank accession number: MN604259). It includes the entire set of 37 genes (i.e. 13 protein-coding genes, 22 tRNA genes and 2 rRNA genes) usually present in animal mitochondrial genomes and a control region. Gene order is identical to that of the putative ancestral arrangement of insects and other brachyceran flies (Boore 1999; Haruyama et al. 2011; Li et al. 2012 [Bibr CIT61052600]; Zhao et al. 2013). There are a total of 35 overlapped nucleotides between genes in 11 locations, ranging from 1 to 8 bp in length; while there are totally 89 bp intergenic nucleotides in 14 locations, ranging from 1 to 19 bp in length.

ATN, GTG, TTG and GTT are accepted canonical mitochondrial start codons for invertebrate mtDNAs and most of PCGs exhibit these start codons (Wolstrnholme 1992). Most of the PCGs in *P. aurifer* mt genome possess common triplet initiation codons ATN (ATC for ND3, ATA for ND1, ATT for ND2, ND5 and ND6, ATG for COII, ATP6, COIII, ND4, ND4L and Cytb), whereas COI starts with CAA and ATP8 with TTG. All of the PCGs stop with complete termination codons (ten with TAA and three with TAG).

The *P. aurifer* mt genome contains 22 tRNA genes ranging from 63 to 72 bp. All the tRNA genes could be folded into a typical cloverleaf secondary structure except for tRNASer (AGN), due to the deficiency of the dihydrouridine (DHU) arm. Among the 22 tRNA genes, 14 are encoded by the H-strand and eight by the L-strand. The lrRNA was 1314 bp in length with an A + T content of 81.2%, while the srRNA is 796 bp long with an A + T content of 77.9%.

The control region is located between srRNA and tRNAIle and is 954 bp in length with an A + T content of 90.7%, which is the most AT-rich region of this mitogenome. The A + T content of the whole genome, PCGs, tRNAs and rRNAs was 76.1%, 74.1%, 75.5%, 79.55%.

Eleven Diptera species were selected to reconstruct a phylogeny with *P. aurifer* (Figure 1). The phylogenetic relationship were estimated using the Neighbor-joining method in MEGA 7. It showed that the phylogenetic relationship of *P. aurifer* was very close to *Hermetia illucens* in the family Stratiomyidae. Meanwhile, the phylogenetic relationship of *P. aurifer* is far away from *Helicoverpa armigera* and *Spodoptera frugiperda*, which are not the species of Order Diptera.

**Figure 1.**
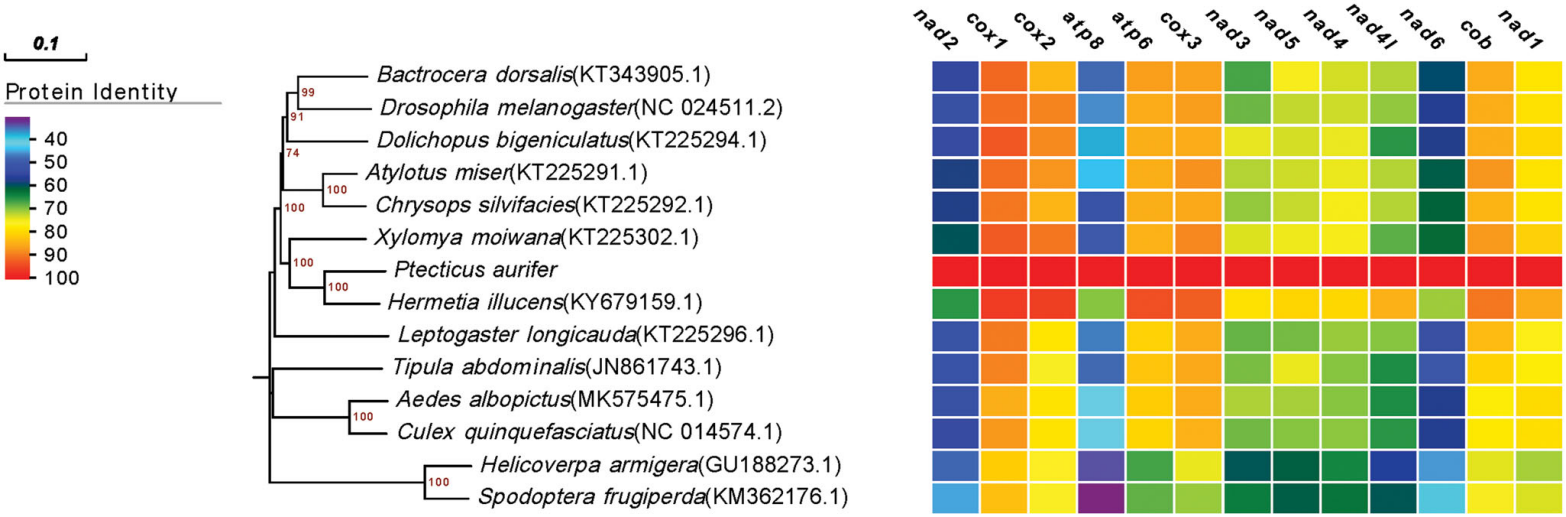
Phylogenetic relationship of 12 Diptera species including Ptecticus aurifer based on the concatenated data set of 13 protein-coding genes. Number above each node indicates the bootstrap support values with 1000 replicates.
